# Occupational Animal Contact in Southern and Central Vietnam

**DOI:** 10.1007/s10393-019-01444-0

**Published:** 2019-11-13

**Authors:** Nguyen Thi Kha Tu, Ngo Tri Tue, Olli Vapalahti, Anna-Maija K. Virtala, Le Van Tan, Maia A. Rabaa, Juan Carrique-Mas, Guy E. Thwaites, Stephen Baker, the VIZIONS consortium

**Affiliations:** 1 Department of Virology, Medicum, University of Helsinki, Helsinki, Finland; 2 The Hospital for Tropical Diseases, Wellcome Trust Major Overseas Programme, Oxford University Clinical Research Unit, 764 Vo Van Kiet, Quan 5, Ho Chi Minh City, Vietnam; 3 Department of Veterinary Biosciences, Faculty of Veterinary Medicine, University of Helsinki, Helsinki, Finland; 4 Virology and Immunology, HUSLAB, Helsinki University Hospital, Helsinki, Finland; 5 Centre for Tropical Medicine and Global Health, Oxford University, Oxford, UK; 6 The Department of Medicine, The University of Cambridge, Cambridge, UK

**Keywords:** Cohort, Emerging infections, Zoonosis, Exposure risk, Vietnam, Slaughterers, Animal health workers

## Abstract

Despite the global zoonotic disease burden, the underlying exposures that drive zoonotic disease emergence are not understood. Here, we aimed to assess exposures to potential sources of zoonotic disease and investigate the demographics, attitudes, and behavior of individuals with sustained occupational animal contact in Vietnam. We recruited 581 animal workers (animal-raising farmers, slaughterers, animal health workers, and rat traders) and their families in southern and central Vietnam into a cohort. Cohort members were followed for 3 years and interviewed annually regarding (1) demography and attitudes regarding zoonotic disease, (2) medical history, (3) specific exposures to potential zoonotic infection sources, and (4) socioeconomic status. Interview information over the 3 years was combined and analyzed as cross-sectional data. Of the 297 cohort members interviewed, the majority (79.8%; 237/297) reported raising livestock; almost all (99.6%; 236/237) reported being routinely exposed to domestic animals, and more than a quarter (28.7%; 68/237) were exposed to exotic animals. Overall, 70% (208/297) reported slaughtering exotic animals; almost all (99.5%; 207/208) reported consuming such animals. The consumption of raw blood and meat was common (24.6%; 73/297 and 37%; 110/297, respectively). Over half (58.6%; 174/297) reported recent occupational animal-induced injuries that caused bleeding; the use of personal protective equipment (PPE) was limited. Our work demonstrates that individuals working with animals in Vietnam are exposed to a wide range of species, and there are limited procedures for reducing potential zoonotic disease exposures. We advocate better education, improved animal security, and enforced legislation of PPE for those with occupational animal exposure in Vietnam.

## Introduction

Zoonoses are infections that can be transmitted from vertebrate animals to humans and vice versa (WHO 2018). Globally, zoonotic infections are responsible for a high disease burden; approximately 60% of all known human diseases and 75% of diseases associated with recent epidemics or pandemics were zoonoses (Woolhouse and Gowtage-Sequeria 2005; Taylor et al. 2009; WHO 2017). Despite the high prevalence of zoonoses, the emergence of zoonotic disease remains difficult to predict and the underlying mechanisms that drive these processes are not well-understood. Studies of zoonotic exposure and hazardous behavior, including the co-sampling of animals, humans, and food products with animal origins, are one approach for better predicting and ultimately intervening in zoonotic disease outbreaks. Contact with infected animals, and/or exposure to contaminated environments, contributes to the emergence and spread of zoonotic diseases in human populations. It is additionally known that increased contact between animals and humans provides more opportunity for exposure to zoonotic pathogens (WHO 2017). Accordingly, the human populations at the highest risk of zoonotic infections are those that have the most frequent interactions with animals. For this reason, slaughterers, animal health workers, animal-raising farmers, and those that trade in wildlife are likely at greater risk of zoonotic infection than those outside of the agricultural industry.

Southeast Asia is considered to be a major hotspot for emerging zoonotic diseases (Morse et al. 2012; Horby et al. 2013). Demography, behavior, attitudes, culture, large animal populations, a high diversity of wild mammalian species, and the coexistence of a broad spectrum of diseases in human and animals are distinctive features of this region, which may lead to the more frequent emergence of zoonotic disease (Morse et al. 2012; Horby et al. 2013; Morand et al. 2014). However, we have a poor understanding of the specific features that lead to zoonotic disease transmission, such as the behavior of those that have sustained contact with animals. Here, we aimed to assess human exposure to animal sources which may be potential reservoirs of zoonotic disease. Additionally, we aimed to investigate the demographics, attitudes, and behavior of assumed high-risk individuals (those with a sustained occupational exposure to animals) living in Vietnam, a country located within the Southeast Asian epicenter of zoonotic diseases. Therefore, we accessed data from a high-risk sentinel cohort (HRSC) study, which was a component of the VIZIONS (Vietnam Initiative on Zoonotic InfectIONS) program (Carrique-Mas et al. 2015; Rabaa et al. 2015) to assess how cohort members interacted with animals and identify potential disease exposure risks.

## Methods

### Ethics

The ethics boards of Dong Thap Hospital, Dak Lak Hospital, the Sub-Departments of Animal Health in Dong Thap and Dak Lak, and the Hospital of Tropical Diseases in Ho Chi Minh City provided ethical approval for this study. The protocol associated with HRSC study was additionally approved by the Oxford Tropical Research Ethics Committee (OxTREC) (No. 157-12) in the UK.

### Study Design

The details of design and implementation of the HRSC study have been described previously (Carrique-Mas et al. 2015). Animal-raising farmers, slaughterers, animal health workers, and rat traders residing in Dong Thap and Dak Lak provinces in the southern and central region of Vietnam, respectively (representing two different geographical and ecological areas), were recruited into the HRSC study (Carrique-Mas et al. 2015; Rabaa et al. 2015). These individuals were broadly representative of people working with animals in rural Vietnamese provinces and were considered to have common occupations associated with continued exposure to animals. Small-scale animal farming is a substantial form of livelihood in these rural provinces and farmers comprise more than two-thirds of the population in the selected areas (GSO—General Statistics Office of Vietnam 2013). Therefore, it was determined that animal-raising farmers should account for approximately two-thirds of the cohort members.

Individuals working in four identified sectors (animal-raising farmers, slaughterers, animal health workers, and rat traders) in selected districts were randomly selected and invited to attend meetings introducing the study. Those with an interest in the study were formally invited to participate; family members of animal farmers were also invited to participate in the study. Animal slaughterers were selected from central slaughter points within each district of the province. Rat traders and animal health workers were selected by convenience. The HRSC study followed the cohort members for three years, starting in June 2013 in Dong Thap province and February 2014 in Dak Lak province. In total, the HRSC was comprised of 581 individuals, including 131 animal-raising farmers, 284 family members of animal-raising farmers, 100 slaughterers, 61 animal health workers, and 5 rat traders. Only adult cohort members working with animals were interviewed (*n* = 297). All cohort members were interviewed on enrollment (first year) and were approached for additional interviews on subsequent years (second and third years); farming households were an exception, only those responsible for raising animals were interviewed.

### Data Collection

The baseline questionnaire used for all participants was comprised of four sections: (1) demography and general information and attitudes regarding animal exposures (2) existing and previous medical history, (3) specific exposures to potential sources of zoonotic infection through primary and secondary occupations, the use of personal protective equipment (PPE) while working with animals, perceived high-risk food-consuming habits, occupational injuries, attitudes to potential exposure risks, and (4) socioeconomic status. The interview data from the first, second, and third year were combined and analyzed as cross-sectional data, resulting in exposure outcomes in at least one of the three interviews.

### Data Preparation and Analysis

Data were prepared by Microsoft Excel (version 2013) and analyzed using STATA statistical software version 12.0. Pearson’s Chi-squared test or Fisher’s exact test was used for pairwise comparisons of categorical variables; the latter when there was a small sample size (< 5) in any of the cells in the contingency table. The Bonferroni method was used for error correction of multiple comparisons (Armstrong 2014). McNemar’s test was used to evaluate the consistency of exposures to animals for the cohort members over the study period. 95% confidence intervals for the percentages/proportions were calculated by the Wilson method (Brown et al. 2001); *P *≤ 0.05 was considered significant. Members of the cohort that were rat traders (*n* = 5) were excluded from analyses of association, difference, or consistency due to an insufficient sample size.

## Results

### Demographic Characteristics

Over the three-year study period, approximately half (51.1%; 297/581) of the cohort members in the two provinces were interviewed on at least one occasion (Table 1). Responses were recorded from 31.6% (131/415) of the animal-raising farmers (one representative on each farm was interviewed) and from all cohort members with other occupations (slaughterers; *n* = 100, animal health workers; *n* = 61, and rat traders; *n* = 5). The median age of those interviewed was 43 years, with an age range of 16–73 years. The majority (53.6%; 156/291; no data from six individuals) of participants had a medium level of education (defined as middle/high-school level), 24.4% (71/291) had a low education level (none/primary level), and 22% (64/291) had a high education level (defined as post-high-school level). 15% (43/291) of participants suffered from at least one underlying chronic disease, including heart disease, diabetes, kidney disease, liver disease, malignancy, lung disease, alcoholism, chronic stomach pain, gastrointestinal disease, and sinusitis.

**Table 1:** General characteristics of animal exposures in the cohort members.

		Province			Occupation				Sex				Age group			
	All	Dong Thap	Dak Lak	*P* value	Farmer	Animal health worker	Slaughterer	*P* value	Female	Male	*P* value	≤ 15	16–44	45–59	≥ 60	*P* value
Study population	581	282	299		415	61	100		259	322		59	308	168	46	
Number interviewed^a^	297	135	162	0.128	131	61	100	< 0.001	93	204	< 0.001	0	163	114	20	0.003
Raising live animals at households or on farms	237 (80)	127 (94)	110 (68)	< 0.001	131 (100)	39 (64)	63 (63)	< 0.001	71 (76)	166 (81)	0.317	0	123 (75)	96 (84)	18 (90)	0.342
Exotic animals	68 (23)	7 (5)	61 (38)	< 0.001	62 (47)	2 (3)	4 (4)	< 0.001	23 (25)	45 (22)	0.611	0	23 (14)	33 (29)	10 (50)	< 0.001
Domestic animals	236 (79)	110 (81)	126 (78)	0.431	130 (99)	39 (64)	63 (63)	< 0.001	71 (76)	165 (81)	0.369	0	123 (75)	95 (83)	18 (90)	0.447
Bleeding injuries while working with animals	174 (59)	70 (52)	104 (64)	0.031	50 (38)	35 (57)	85 (85)	< 0.001	51 (55)	123 (60)	0.376	0	106 (65)	62 (54)	6 (30)	0.018
Slaughtering/cooking and consuming exotic animals	208 (70)	58 (43)	150 (93)	< 0.001	90 (69)	42 (69)	71 (71)	1	66 (71)	142 (70)	0.812	0	117 (72)	79 (69)	12 (60)	1
Slaughtering/cooking	65 (22)	22 (16)	43 (27)	0.033	40 (31)	10 (16)	11 (11)	0.003	16 (17)	49 (24)	0.188	0	36 (22)	23 (20)	6 (30)	1
Consuming	207 (70)	57 (42)	150 (93)	< 0.001	90 (69)	42 (69)	70 (70)	1	67 (72)	140 (69)	0.553	0	115 (71)	79 (69)	12 (60)	1
Consumed raw blood or meat	139 (47)	56 (41)	83 (51)	0.093	64 (49)	31 (51)	39 (39)	0.678	22 (24)	117 (57)	< 0.001	0	87 (53)	41 (36)	11 (55)	0.039
Raw blood	73 (25)	7 (5)	66 (41)	< 0.001	32 (24)	16 (26)	25 (25)	1	8 (9)	65 (32)	< 0.001	0	44 (27)	24 (21)	5 (25)	1
Raw meat	110 (37)	53 (39)	57 (35)	0.469	51 (39)	23 (38)	31 (31)	1	16 (17)	94 (46)	< 0.001	0	69 (42)	31 (27)	10 (50)	0.051

The values are shown in format of number (percentage). The denominator for the percentages is the value on the top row.

^a^Cohort members interviewed at least once among three baseline interviews (*n* = 297), including at enrollment (first year, 2013–2014, *n* = 291), second (2014–2015, *n* = 273) and third year (2015–2016, *n* = 265) in the high-risk sentinel cohort (HRSC) study (*n* = 581), cohort members interviewed on all three occasion (*n* = 252).

### Exposure to Live Animals

Of the 297 cohort members interviewed on at least one occasion, the majority (79.8%; 237/297) reported raising livestock either in their backyard, in the area surrounding their household, or on adjoining farmland (Table 2). 63.9% (95% CI 56.3–70.8%; 106/166) of the interviewed non-professional farmers also reported raising animals (i.e., small-scale backyard farming) (Table 2). Almost all (99.6%; 236/237) of the cohort members reported being routinely exposed to domestic animals, and over a quarter (28.7%; 68/237) were exposed to exotic (non-domestic) animals. The most common exotic animal exposures were wild pigs (61.8%; 42/68), wild birds (30.9%; 21/68), deer (20.6; 14/68), and porcupines (16.2%; 11/68). Dogs (85.2%; 201/236), chickens (79.2%; 187/236), pigs (53.8%; 127/236), and cats (53.8%; 127/236) were the most common domestic animals that cohort members were exposed to.

**Table 2:** Exposure to live animals in the cohort.

	Occupational exposures (*n* = 131)	Non-occupational exposures (*n* = 166)			Total	95% CI
	Farmers	Animal health workers	Slaughterers	Rat traders		
Interviewed cohort members^a^*N*	131	61	100	5	297	
Raising reported^d^	131 (100.0)	39 (63.9)	63 (63.0)	4 (80.0)	237 (79.8)	74.9–84.0
Raising of exotic animals	62 (47.3)	2 (5.1)	4 (6.3)		68 (28.7)	23.3–34.8
Wild pig	37 (28.2)	2 (5.1)	3 (4.8)		42 (61.8)	49.9–72.4
Other wild birds^b^	17 (13.0)	2 (5.1)	2 (3.2)		21 (30.9)	21.2–42.6
Deer	14 (10.7)				14 (20.6)	12.7–31.6
Porcupine	11 (8.4)				11 (16.2)	9.3–26.7
Jungle fowl	5 (3.8)				5 (7.4)	3.2–16.1
Monkey	2 (1.5)				2 (2.9)	0.8–10.1
Civet	2 (1.5)				2 (2.9)	0.8–10.1
Bamboo rat^c^	1 (0.8)	1 (2.6)			2 (2.9)	0.8–10.1
Bat	1 (0.8)				1 (1.5)	0.3–7.9
Pheasant	1 (0.8)				1 (1.5)	0.3–7.9
Raising of domestic animals^e^	130 (99.2)	39 (100.0)	63 (100.0)	4 (100.0)	236 (99.6)	97.7–99.9
Dog	118 (90.8)	35 (89.7)	44 (69.8)	4 (100.0)	201 (85.2)	80.1–89.1
Chicken	128 (98.5)	26 (66.7)	31 (49.2)	2 (50.0)	187 (79.2)	73.6–83.9
Pig	87 (66.9)	18 (46.2)	22 (34.9)		127 (53.8)	47.4–60.1
Cat	83 (63.8)	18 (46.2)	23 (36.5)	3 (75.0)	127 (53.8)	47.4–60.1
Duck	51 (39.2)	4 (10.3)	14 (22.2)	2 (50.0)	71 (30.1)	24.6–36.2
Muscovy duck	51 (39.2)	3 (7.7)			54 (22.9)	18.0–28.7
Pigeon	25 (19.2)	5 (12.8)	3 (4.8)		33 (14.0)	10.1–19.0
Cattle	15 (11.5)	5 (12.8)	10 (15.9)		30 (12.7)	9.1–17.6
Goose	21 (16.2)	1 (2.6)	3 (4.8)		25 (10.6)	7.3–15.2
Goat	12 (9.2)	2 (5.1)	2 (3.2)		16 (6.8)	4.2–10.7
Rabbit	15 (11.5)				15 (6.4)	3.9–10.2
Buffalo	1 (0.8)		5 (7.9)		6 (2.5)	1.2–5.4
Quail	2 (1.5)				2 (0.8)	0.2–3.0
Turkey	2 (1.5)				2 (0.8)	0.2–3.0

^a^The cohort members interviewed at least once among three baseline interviews, including at enrollment (first year), second and third years. The values are shown in format of number (percentage: 95% CI). The empty cells equal to “0”.

^b^Other wild birds than pheasants.

^c^*Rhizomys sumatrensis.*

^d^Denominators of analyses of raising of any exotic animals groups.

^e^Denominators of corresponding subgroups analyses.

There was a significant difference in exposure to exotic animals by occupation, age group, level of education, and area of residence. Notably, farmers (45.8%; 60/131) were significantly more commonly exposed to exotic animals than slaughterers (4%; 4/100) and animal health workers (3.3%; 2/61) (*P *< 0.001) (Table 1). Similarly, cohort members in the 60 + (50%; 10/20) and 45–59 year age groups (29%; 33/114) were more regularly exposed to exotic animals than those in the 16–44 year age group (14.1%; 23/163) (*P *< 0.001). Cohort members in Dak Lak were significantly more exposed to exotic animals than cohort members in Dong Thap; 37.7% (61/162) and 5.2% (7/135) (*P *< 0.001), respectively.

### Animal Exposure in Animal Health Workers and Slaughterers

Animal slaughterers (*n* = 100) and animal health workers (*n* = 61) were exposed to 17 different animal species; the most common were chickens, ducks, Muscovy ducks, pigs, and cattle (Table 3). We observed a significant difference in animal exposure by area of residence. Animal health workers residing in Dak Lak were more frequently exposed to beef cattle than those in residing in Dong Thap (*P *= 0.024). Alternatively, animal health workers in Dong Thap were more commonly exposed to Muscovy ducks than animal health workers in Dak Lak (*P *= 0.013). Furthermore, slaughterers in Dak Lak were more commonly exposed to beef cattle, buffaloes, and geese than those residing in Dong Thap (*P *= 0.001, 0.014, and 0.001, respectively) (Table 3).

**Table 3:** Animals exposures in slaughterers and animal health workers.

	Slaughterers			Animal health workers		
	Dong Thap	Dak Lak	Total	Dong Thap	Dak Lak	Total
Interviewed cohort members	33	67	100	30	31	61
Reported exposure, *N*	33	67	100	30	31	61
Chicken	18 (54.5)	35 (52.2)	53 (53.0: 43.3–62.5)	29 (96.7)	31 (100.0)	60 (98.4: 91.3–99.7)
Duck	18 (54.5)	35 (52.2)	53 (53.0: 43.3–62.5)	29 (96.7)	26 (83.9)	55 (90.2: 80.2–95.4)
						(*P *= 0.013)
Muscovy duck	18 (54.5)	30 (44.8)	48 (48.0: 38.5–57.7)	22 (73.3)	13 (41.9)	35 (57.4: 44.9–69.0)
Pig	15 (45.5)	31 (46.3)	46 (46.0: 36.6–55.7)	29 (96.7)	31 (100.0)	60 (98.4: 91.3–99.7)
Cattle		16 (23.9)	16 (16.0: 10.1–24.4)	25 (83.3)	31 (100.0)	56 (91.8: 82.2–96.5)
			(*P *= 0.001)			(*P *= 0.024)
Geese		16 (23.9)	16 (16.0: 10.1–24.4)	8 (26.7)	5 (16.1)	13 (21.3: 12.9–33.1)
			(*P *= 0.001)			
Buffalo		11 (16.4)	11 (11.0: 6.3–18.6)	6 (20.0)	11 (35.5)	17 (27.9: 18.2–40.2)
			(*P *= 0.014)			
Rabbit		8 (11.9)	8 (8.0: 4.1–15.0)			
Pigeon		2 (3.0)	2 (2.0: 0.6–7.0)			
Cat		1 (1.5)	1 (1.0: 0.2–5.5)	8 (26.7)	1 (3.2)	9 (14.8: 8.0–25.7)
Rice field rat	1 (3.0)		1 (1.0: 0.2–5.5)			
Dog		1 (1.5)	1 (1.0: 0.2–5.5)	21 (70.0)	10 (32.3)	31 (50.8: 38.6–62.9)
Goat					8 (25.8)	8 (13.1: 6.8–23.8)
Porcupine				1 (3.3)	2 (6.5)	3 (4.9: 1.7–13.5)
Wild pig				1 (3.3)	1 (3.2)	2 (3.3: 0.9–11.2)
Monkey				1 (3.3)		1 (1.6: 0.3–8.7)
Other wild bird				1 (3.3)		1 (1.6: 0.3–8.7)

The values are shown in format of number (percentage: 95% CI). Empty cells equal to “0.” Statistically significant differences between variables at 5% level are shown.

### Slaughtering, Cooking and Consuming Exotic Animals

Of all the interviewed cohort members, 70% (208/297) reported slaughtering, cooking, or consuming exotic animals within the year prior to interview (Table 1). The majority (99.5%; 207/208) reported consuming such animals, and 31.3% (65/208) reported slaughtering or cooking these animals. The most common exotic animals that the cohort members were exposed to through slaughtering, cooking, or consuming were wild pigs, deer, and porcupines (Table 4). These animals were generally raised in their backyards, the area surrounding their own household, or on specific wildlife farms (Table 2). Porcupines, civets, deer, jungle fowl, squirrels, and pangolins were slaughtered/cooked in Dak Lak only. Correspondingly, only participants in Dak Lak reported consuming civets, squirrels, jungle fowl, pangolins, and wild rabbits (Table 4). Cohort members in Dak Lak were significantly more likely to slaughter, cook, and consume exotic animals than those in Dong Thap (26.5% (43/162) vs. 16.3% (22/135); and 92.6% (151/163) vs. 41.5% (56/135), respectively; *P *= 0.033 and *P *< 0.001, respectively) (Table 1).

**Table 4:** Exposure to exotic animals by slaughtering, cooking, or consuming.

		Farmers	Animal health workers	Slaughterers	Rat traders	Total
Interviewed cohort members, N		131	61	100	5	297
Reported exposure^a^		90 (68.7)	42 (68.9)	71 (71.0)	5 (100.0)	208 (70.0: 64.6–75)
Slaughtering and cooking	All exposed^b^	39 (43.3)	10 (23.8)	11 (15.5)	5 (100.0)	65 (31.3: 25.3–37.8)
	Wild pig	25 (64.1)	6 (60.0)	3 (27.3)		34 (52.3: 40.4–64.0)
	Rice field rat	4 (10.3)	5 (50.0)	7 (63.6)	5 (100.0)	21 (32.3: 22.2–44.4)
	Porcupine	8 (20.5)	1 (10.0)			9 (13.8: 7.5–24.3)
	Civet	2 (5.1)	2 (20.0)			4 (6.2: 2.4–14.8)
	Bamboo rat	1 (2.6)	1 (10.0)	1 (9.1)	1 (20.0)	4 (6.2: 2.4–14.8)
	Deer	2 (5.1)	2 (20.0)			4 (6.2: 2.4–14.8)
	Jungle fowl	3 (7.7)				3 (4.6: 1.6–12.7)
	Squirrel	2 (5.1)				2 (3.1: 0.9–10.5)
	Pangolin	1 (2.6)				1 (1.5: 0.3–8.2)
	Other wild bird	1 (2.6)				1 (1.5: 0.3–8.2)
Consuming	All exposed^c^	90 (100.0)	42 (100.0)	70 (98.6)	5 (100.0)	207 (99.5: 97.3–99.9)
	Wild pig	65 (72.2)	31 (73.8)	56 (80.0)		174 (73.4: 78.5–88.4)
	Deer	38 (42.2)	16 (38.1)	18 (25.7)		72 (34.8: 28.6–41.5)
	Porcupine	31 (34.4)	17 (40.5)	16 (22.9)		64 (30.9: 25.0–37.5)
	Rice field rat	25 (27.8)	15 (35.7)	12 (17.1)	4 (80.0)	56 (27.1: 21.5–33.5)
	Civet	9 (10.0)	7 (16.7)	4 (5.7)		20 (9.7:6.3–14.5)
	Bamboo rat	4 (4.4)	4 (9.5)	2 (2.9)	1 (20.0)	11 (5.3: 3.0–9.3)
	Jungle fowl	6 (6.7)	1 (2.4)	1 (1.4)		8 (3.9: 2.0–7.4)
	Squirrel	2 92.2)	2 (4.8)			4 (1.9: 0.8–4.9)
	Other wild bird	3 (3.3)	1 (2.4)			4 (1.9: 0.8–4.9)
	Pangolin	1 (1.1)		1 (1.4)		2 (1.0: 0.3–3.5)
	Bat	2 (2.2)				2 (1.0: 0.3–3.5)
	Monkey	2 (2.2)				2 (1.0: 0.3–3.5)
	Wild rabbit		1 (2.4)			1 (0.5: 0.1–2.7)

At least once among three baseline interviews, including at enrollment (first year), second and third years. The values are shown in format of number (percentage: 95% CI). The empty cells equal to “0”.

^a^Denominators of analyses of “All exposed” groups.

^b^Denominators of analyses of “Slaughtering and cooking” groups.

^c^Denominators of analyses of “Consuming” groups.

### Consuming of Raw Animal Blood or Raw Meat

Raw animal blood is commonly consumed in Vietnam as a dish named “tiet canh.” Almost a quarter (24.6%; 73/297) of interviewed cohort members reported the consumption of raw blood, and over a third (37%; 110/297) had consumed raw mammal or bird meat within the year prior to interview (Table 5). Cohort members typically reported consuming raw blood 1–3 times per year (72.6%; 53/73), and over a half (53.6%; 59/110) reported eating raw meat ≥ 4 times per year. The most commonly consumed animal blood was (sequentially) from pigs (40.1%; 61/152), ducks (38.2%; 58/152), rabbits (7.2%; 11/152), Muscovy ducks (5.9%; 9/152), goats (3.9%; 6/152), deer (1.3%; 2/152), and beef cattle (1.3%; 1/152). The most commonly consumed raw meat was from beef cattle (91%; 142/156); the consumption of raw meat from pigs (5.1%; 8/156), goats, rabbits, chickens, and quails were less common (< 2% each, data not shown). Cohort members in Dak Lak reported consuming raw blood more commonly than those in Dong Thap (40.7% (66/162) vs. 5.2% (7/135), respectively, *P *< 0.001) (Table 1). Men were more likely to consume raw blood (31.9% (65/204) or raw meat (46.1%; 94/204) than women (8.6% (8/93), *P *< 0.001 and 17.2% (16/93), *P *< 0.001, respectively). The majority (61.6%; 45/73) of raw-blood consumers considered this activity to be not good for their health, the remainder thought it was healthy, were not sure, or had no opinion (38.3%; 28/73) (Table 5). Regarding the consumption of raw animal meat, 33.6% (37/110) of consumers acknowledged that it was probably not good for their health, while the majority (51.8%; 57/110) thought that eating raw meat was good for their health.

**Table 5:** Raw-blood and raw-meat consumption.

	Farmer	Animal health worker	Slaughterer	Rat trader	Total
Interviewed cohort members, *N*	131	61	100	5	297
Reported consumption	64 (48.9)	31 (50.8)	62 (62.0)	5 (100.0)	162 (54.5: 48.9–60.1)
Raw-blood consumption					
None	99 (75.6)	45 (73.8)	75 (75.0)	5 (100.0)	224 (75.4: 70.2–80.0)
Yes^a^	32 (24.4)	16 (26.2)	25 (25.0)		73 (24.6: 20.0–29.8)
1–3 times	21 (65.6)	15 (93.8)	17 (68.0)		53 (72.6: 61.4–81.5)
≥ 4 times	11 (34.4)	1 (6.3)	8 (32.0)		20 (27.4: 18.5–38.6)
Opinion about raw-blood consumption					
Good	4 (12.5)	1 (6.3)	8 (32.0)		13 (17.8: 10.7–28.1)
Not good	23 (71.9)	12 (75.0)	10 (40.0)		45 (61.6: 50.2–72.0)
No opinion or not sure	5 (15.6)	3 (18.8)	7 (28.0)		15 (20.5: 12.9–31.2)
Raw-meat consumption					
None	80 (61.1)	38 (62.3)	69 (69.0)		187 (63.0: 57.3–68.3)
Yes^b^	51 (38.9)	23 (37.7)	31 (31.0)	5 (100.0)	110 (37.0: 31.7–42.7)
1–3 times	25 (49.0)	9 (39.1)	13 (41.9)	4 (80.0)	51 (46.4: 37.3–55.7)
≥ 4 times	26 (51.0)	14 (60.9)	18 (58.1)	1 (20.0)	59 (53.6: 44.4–62.7)
Opinion about raw-meat consumption					
Good	28 (54.9)	10 (43.5)	18 (58.1)	1 (20.0)	57 (51.8: 42.6–60.9)
Not good	18 (35.3)	10 (53.5)	9 (29.0)		37 (33.6: 25.5–42.9)
No opinion or not sure	5 (9.8)	3 (13.0)	4 (12.9)	4 (80.0)	16 (14.5: 9.2–22.3)

At least once among three baseline interviews, including at enrollment (first year), second and third years, and their opinions about the consumption. The values are shown in format of number (percentage: 95% CI). The empty cells equal to “0”.

^a^Denominators of analyses of “raw-blood consumption” frequency (1–3 times and ≥ 4 times) and “opinions about raw-blood consumption” opinions (Good, Not good or No opinion or not sure).

^b^Denominators of analyses of “raw-meat consumption” frequency (1–3 times and ≥ 4 times) and “opinions about raw-meat consumption” opinions (good, not good, or no opinion or not sure).

### Bleeding and Biting Injuries

Over half (58.6%; 174/297) of the 297 interviewed cohort members reported recent occupational injuries that induced bleeding while working with the animals (Table 6). The majority of these cohort members (70.1%; 122/174) reported being injured 1–3 times per year; more than a quarter (29.9%; 52/174) reported being injured ≥ 4 times a year. Cohort members were most frequently bitten by pigs, chicken, ducks, beef cattle, buffalo, wild pigs, dogs, and rats. Other bleeding injuries induced by working with the animals were associated with knives, needles, and skin abrasions. Overall, cohort members in Dak Lak (64.2%; 104/162) were injured more frequently than those in Dong Thap (51.9%; 70/135) (*P *= 0.031). Slaughterers (85%; 85/100) experienced significantly more bleeding injuries than animal health workers (57.4%; 35/61) and farmers (38.2%; 50/131) (*P *< 0.001). Members in the 60 + year age group (30%; 6/20) were less regularly injured than those in the 45–59 (54.4%; 62/114) and 16–44 year age groups (65%; 106/163) (*P *= 0.018) (Table 1).

**Table 6:** Bleeding and biting injuries when working with animals.

		Farmers	Animal health workers	Slaughters	Rat traders	Total
Interviewed cohort members, *N*		131	61	100	5	297
Reported injuries*		50 (38.2)	35 (57.5)	85 (85.0)	4 (80.0)	174 (58.6: 52.9–64.0)
Bleeding injuries	Bitten	22 (44.0)	21 (60.0)	9 (10.6)	3 (75.0)	55 (31.6: 25.2–38.9)
	Other injuries	36 (72.0)	29 (82.9)	85 (100.0)	4 (100.0)	154 (88.5: 82.9–92.4)
	1–3 times	41 (82.0)	30 (85.7)	51 (60.0)		122 (70.1: 62.9–76.4)
	≥ 4 times	9 (18.0)	5 (14.3)	34 (40.0)	4 (100.0)	52 (29.9: 23.6–37.1)

At least once among three baseline interviews, including at enrollment (first year), second and third years. The values are shown in format of number (percentage: 95% CI). The empty cells equal to “0”.

^a^Denominators of subsequent analyses of corresponding variables/groups.

### The Use of Personal Protective Equipment

Over two-thirds (69/100) of slaughterers at abattoirs reported never using PPE, and only one worker reported using full PPE. When used, gloves were the most common piece of PPE, followed by boots, face masks, and aprons. In contrast, < 5% of slaughters reported using a mob caps/hats or goggles (Table 7). We found that animal slaughterers in Dong Thap reported not using PPE (93.9%; 31/33) more commonly than animal slaughterers in Dak Lak (56.7%; 38/67) (*P *< 0.001) (Table 7). There was a significant association between those reported being bitten by animals and those using PPE; slaughters not using any PPE were bitten to the point of bleeding (13.04%; 9/69) more commonly than those reporting the use any piece of PPE (0%; 0/69) (*P *= 0.054).

**Table 7:** The use of personal protective equipment (PPE) at abattoirs.

	Dong Thap	Dak Lak	Total
Interviewed cohort members *N*	33	67	100
No usage of any piece of PPE	31 (93.9)	38 (56.7)	69.0: 59.4–77.2)
			(*P *< 0.001)
Full PPE		1 (1.5)	1 (1.0: 0.2–5.5)
Gloves	5 (15.2)	59 (88.1)	64 (64.0: 54.2–73.7)
Boots	5 (15.2)	54 (80.6)	59 (59.0: 49.2–68.1)
Face mask	6 (18.2)	50 (74.6)	56 (56.0: 46.2–65.3)
Apron		22 (31.8)	22 (22.0: 15.0–31.1)
Hat/mob cap		4 (6.0)	4 (4.0: 1.6–9.8)
Goggles		2 (3.0)	2 (2.0: 0.6–7.0)

At least once among three baseline interviews, including at enrollment (first year), second and third years. Each separate piece of PPE indicates that at least this PPE was used. The values are shown in format of number (percentage: 95% CI). The empty cells equal to “0.” The statistically significant differences between variables at 5% level are shown.

Despite most cohort members reporting direct contact with animals on a daily basis, one-fifth (20.3%; 59/291) reported doing nothing, did not answer, or did not know what to do when bitten. Almost a quarter (22%; 64/291) reported using no gloves, facemasks, or protective clothing when routinely touching animals. Additionally, over two-thirds of members (68.7%; 200/291) thought, or did not know, that they could not get an infection from having contact with apparently healthy animals. Over a quarter (28.2%; 82/291) of the cohort members thought they could not contract an infection through direct contact with diseased animals.

### Exposure Consistency Over the Study Period

Over a half (51.1%; 297/581) of the cohort members were interviewed at least once, and 84.8% (252/297) of the cohort members were interviewed on all three occasions; 89.2% (265/297) members were interviewed at year three. The reporting of direct animal exposures reported was consistent over the three-year study period (*P *> 0.05). However, the consumption of raw animal blood declined significantly between year one and year two versus year three (17.9% (45/251) vs. 8.1% (20/231), *P *= 0.0005), and (13.8% (35/254) vs. 7.9% (20/254), *P *= 0.01, respectively). The same trend between year one and year three was observed for raw-meat consumption (22.3% (58/260) vs. 16.2% (42/260), *P *= 0.048). Additionally, slaughters reported using PPE more commonly in the first year than the third year (57.3% (51/89) vs. 32.6% (29/89), *P *= 0.0001). Overall, cohort members reported getting bitten or other animal-induced injuries significantly less in year two and three than year one (bitten: 3.3% (9/269) vs. 17.1% (46/269), *P *< 0.0001 and 3.1% (8/261) vs. 17.2% (45/261), *P *< 0.0001, respectively) (other injuries: 20.9% (56/268) vs. 38.1% (102/268), *P *< 0.0001 and 18.8 (49/260) vs. 38.1% (99/260), *P *< 0.0001, respectively).

## Discussion

Our results indicate that, besides their own occupational exposures, cohort members in the selected locations were regularly exposed to a large variety of differing animals. Farmers were the most commonly interviewed group; therefore, we unsurprisingly found that the principal animal exposures in this population came from raising livestock. More than three quarters of all interviewed participants (animal-raising farmers, slaughterers, animal health workers, and rat traders) and over two-thirds of the interviewed subjects, with the exception of animal-raising farmers, reported raising exotic or domestic animals in their backyard or around the family household. Exposure to exotic animals was greater in Dak Lak province than in Dong Thap province, which largely reflects the distinct profiles of the two locations. Dong Thap is closer to Ho Chi Minh City; therefore, there is a greater demand for non-exotic meat. In contrast, Dak Lak is more remote and caters more for the local rural population.

Beside routine occupational exposures to animals, cohort subjects had contact with a large variety of animal types, including fifteen types of exotic and domestic animals. Moreover, the animal species that the cohort members were most exposed to (wild pigs, porcupines, rice field rats, deer, pigs, chickens, dogs, cats, ducks, and cattle) are known to be potential reservoirs for zoonotic pathogens (Hart and Trees 1997; Acha and Szyfres 2003; Meng et al. 2009; Kreuder Johnson et al. 2015). These interactions with a range of animals are related to the fact that more than two-thirds of those in the cohort practiced backyard farming. This proportion was higher than the average within the Vietnamese population; approximately 50% of all households in Vietnam are estimated to farm animals (GSO—General Statistics Office of Vietnam 2013), and small-scale animal production is particularly common in rural Vietnam. This industry is maintained by the higher prices (often more than double) demanded for “home reared” animal meat in comparison to animals raised in industrial facilities. Additionally, rural Vietnamese people like to support their community and purchase local produce. A large range of activates in this industry is highlighted by the fact that the majority of farming families reported raising several animal species in small numbers in or around their households (Phuong et al. 2015). These data suggest a low level of biosecurity with the potential for the mixing of multiple animal species; we speculate that this increases the risk of pathogen transfer between animals and may lead to a greater likelihood for exposure to zoonotic diseases in those farming animals. The presence of a large variety of animal types in small areas, particularly in single households farming a mixture of animal species, may increase opportunity for species-crossing pathogen transmission events, as illustrated by the emergence of avian influenza H5N1 virus in Vietnam in 2003, with the first reported case coming from Dong Thap.

We found that safety procedures for those handling live animals or involved in slaughtering or butchering were inadequate. Notably, a low proportion of cohort members reported not using any PPE when handling animals. These data indicate a pervasive poor understanding of occupational exposure that may result in increased potential for zoonotic disease transmission if animals are infected with zoonotic pathogens. This lack of PPE usage was specifically common among the animal slaughterers, where contact with fresh blood is a sustained occupational hazard. The risk of such activities is highlighted by recent reports of the transmission of *Trypanosoma evansi* and rabies in central and northern Vietnam, which likely occurred during the butchering of raw meat, and processing of the animal brain or via contact with animal saliva, respectively (Wertheim et al. 2009b; Chau et al. 2016). A lack of PPE was also significantly associated with being bitten by animals; while this is not surprising, the proportion of other animal-induced injuries also corresponded with low PPE usage. A study conducted by Rui Carlos and co-workers (Schneider et al. 2013) found that a lack of PPE usage in Brazil was associated with the transmission of *Brucella abortus* from animals to slaughterers at slaughterhouses. Therefore, this lack of PPE usage may indicate a higher exposure risk for zoonotic infections among cohort subjects. This observation is particularly concerning, and we advocate better education for PPE use in animal worker and slaughters. We suggest that these workers are a key population for exposure to common zoonotic pathogens, such as *Brucella,* which has been recently found endemic in Vietnam and poses a major risk to human health (Campbell et al. 2017). Additional follow-up studies encompassing serological testing are required to test this hypothesis.

In Vietnam, animal-product consumers enjoy many foods that would be considered atypical in the west; exotic animals are a particular delicacy. Almost all of the interviewed cohort members had consumed at least one of thirteen different types of exotic animal over the three-year study period. The thought is that the consumption of exotic animals has preventative health benefits and/or a positive medical treatment effect. For example, the consumption of porcupine stomach is believed to cure stomach pain, and porcupine bile is used as an analgesic. Furthermore, grated deer horn is thought to treat a multitude of chronic diseases and prolong life. A general increase in income across the population and permission from the Vietnamese government for wildlife farming in recent years (Ministry of Agriculture and Rural Development—Vietnam) have played a major role in the rise of exotic animal consumption. The overlap between the types of animals raised, slaughtered, cooked, and consumed by the cohort subjects indicates that increasing consumption requires more intensive farming, slaughtering, cooking, and the hunting exotic animals to supply this increasing demand. As the current economic trajectory is predicted to continue to improve across Vietnam (The World Bank in Vietnam 2017), it is probable that the consumption of exotic animals will increase. This increase in the rearing intensity of exotic animals may have a possible knock-on effect for disease exposures within the general population.

An additional specific practice in Vietnam that may increase the risk of zoonotic exposures is consumption of raw animal blood or meat. More than a half of the cohort members reported the consumption of raw animal blood or meat over the study period. This proportion was high, especially considering that sale of raw blood dishes was banned by the Vietnamese government in 2009 (The Agriculture Ministry of Vietnam). The consumption of raw animal blood is found to have a higher possibility of infections from blood-borne zoonotic pathogens such as *Streptococcus suis* (Wertheim et al. 2009a; Navacharoen et al. 2009; Trung et al. 2011), *Trichinella spiralis* (Taylor et al. 2009; De et al. 2015), and *Brucella* spp. (Njeru et al. 2016). Counter-intuitively, we also found that the majority of the raw-blood consumers considered that eating raw blood was not good for their health. Raw-blood consumption in Vietnam is largely cultural and is a common dish at special gatherings or celebrations in several geographic areas. Raw animal blood is also thought to have potential health benefits, such as boosting the immune system, reducing body temperature, preventing anemia, and as treating headaches, coughs, and dysentery (Thi et al. 2014). These social factors show that culture strongly drives consumer tastes, and restricting the population eating raw animal blood is more of a social issue than a scientific one.

An analysis of demography in the cohort members further demonstrated potential risks of zoonotic disease exposure. Approximately a quarter of the subjects had a low education level, which has been previously associated with a higher incidence of numerous communicable diseases (Zimmerman and Woolf 2014; Bruce et al. 2019). These data, together with a high exposure to potential sources of zoonotic disease, indicate a possible elevated risk of zoonotic infections in the cohort subjects. We also identified significant differences between exposures to potential zoonotic sources by location, sex, age group, education level, and profession; high frequency of contact with animals is associated with a likelihood of increased zoonotic transmission (Howard and Fletcher 2012; WHO 2017). Therefore, it is important to elucidate the demographic/social factors that drive zoonotic infections to induce feasible interventions and to determine whether consistency or variation in exposure over time results elevated or reduced risk. To achieve this, it will be essential to further gather information on the incidence of zoonotic disease in those that work with animals and also measure the exposure to given pathogens.

Our study contains limitations. This was a cohort consisting of individuals with a perceived risk of zoonotic disease, and control subjects without animal exposures were not recruited. Additionally, we did not distinguish between those farming or hunting exotic animals, as raising many types of exotic animals is common and permitted in Vietnam. Furthermore, we did not evaluate disease episodes with a suitable control group (those not working with animals) or measure serological exposure; therefore, we cannot estimate whether those working with animals do have an increased incidence of infectious disease from an animal origin.

Despite the apparent limitations, our study illustrates that, in addition to occupational exposures, individuals that work with animals in Vietnam are frequently exposed to a range of animal species which is likely to increase their risk of zoonotic disease exposures. Sustained animal exposure and a large variety of animal species demonstrate that slaughterers, animal health workers, animal-raising farmers, and rat traders are sentinel professions for performing zoonotic disease surveillance. Additionally, the attitudes and behavior of the cohort members show that they have limited knowledge of potentially zoonotic disease exposures. The data presented here, in combination with further sero-epidemiological and molecular studies, will aid in elucidating the potential factors associated with a comparatively high incidence of emerging zoonotic disease in Southeast Asia. Ultimately, our findings will be useful for better preparedness, intervention plans, disease prediction models, and the development of future research into zoonotic infections in Southeast Asia.
